# Data Fusion Combining High-Resolution Mass Spectrometry and ^1^H-NMR Metabolomic Data with Gluten Protein Content to Assess the Impact of Agro-Sustainable Treatments on Durum Wheat

**DOI:** 10.3390/molecules31060922

**Published:** 2026-03-10

**Authors:** Nicolò Riboni, Enmanuel Cruz Muñoz, Christina Muhs, Monica Mattarozzi, Marina Caldara, Sara Graziano, Christian Richter, Harald Schwalbe, Nelson Marmiroli, Davide Ballabio, Mariolina Gullì, Maria Careri, Federica Bianchi

**Affiliations:** 1 Department of Chemistry, Life Sciences and Environmental Sustainability, University of Parma, Parco Area delle Scienze 11/A-17/A, 43124 Parma, Italy; monica.mattarozzi@unipr.it (M.M.); marina.caldara@unipr.it (M.C.); sara.graziano@unipr.it (S.G.); nelson.marmiroli@unipr.it (N.M.); maria.careri@unipr.it (M.C.); 2 Milano Chemometrics and QSAR Research Group, Department of Earth and Environmental Sciences, University of Milano-Bicocca, Piazza della Scienza 1, 20126 Milan, Italy; enmanuel.cruzmunoz@unimib.it (E.C.M.); davide.ballabio@unimib.it (D.B.); 3 Center of Biomolecular Magnetic Resonance (BMRZ), Goethe University Frankfurt, Max-von-Laue-Straße 9, 60438 Frankfurt am Main, Germany; muhs@nmr.uni-frankfurt.de (C.M.); ric@nmr.uni-frankfurt.de (C.R.); schwalbe@nmr.uni-frankfurt.de (H.S.)

**Keywords:** untargeted metabolomics, data fusion, high-resolution mass spectrometry, nuclear magnetic resonance spectroscopy, sustainable treatments, durum wheat

## Abstract

Sustainable food production systems based on the use of biofertilizers and soil improvers are proposed to mitigate agricultural-related environmental impacts and address the climate crisis. In particular, plant growth-promoting microbes (PGPM) and biochar (Char) have been reported to improve plant growth, soil quality, and crop yield; however, their effects on food quality remain debated. In this study, untargeted metabolomics based on ultra-high performance liquid chromatography–ion mobility–high-resolution mass spectrometry (UHPLC-IMS-HRMS) and proton nuclear magnetic resonance spectroscopy (^1^H-NMR) are proposed to achieve a comprehensive investigation of the effects of Char, PGPM and Char+PGPM on durum wheat. A total of 88 metabolites were annotated by UHPLC-IMS-HRMS, mainly belonging to carbohydrates, flavones, flavonoids, glycerophospholipids, and glycolipids, while 30 compounds were annotated by ^1^H-NMR, mostly amino acids and short-chain carboxylic acids. The two datasets were merged with the gluten protein content dataset by using low- and mid-level data fusion approaches, obtaining models that exhibit excellent classification performance. Integrated analysis highlighted that the combined Char+PGPM treatment induced metabolic changes across multiple chemical classes, including enrichment of flavonoids and lipids, and downregulation of carbohydrate metabolites, suggesting a redistribution of carbon resources and modulation of secondary metabolism with potential implications on wheat grain quality.

## 1. Introduction

The global food system is under increasing pressure to meet the nutritional demand of a growing population while simultaneously addressing climate changes, minimizing environmental impacts, promoting sustainable agricultural practices, and ensuring food safety and security [1,2,3]. Although fertilizers play a crucial role in increasing crop yields, their excessive use has significantly contributed to environmental pollution, soil degradation, and the accumulation of harmful chemical residues in the food chain, thus threatening ecosystems and raising serious concerns for human health. In response, the shift toward more sustainable agricultural practices has driven a growing interest in environmentally friendly alternatives to synthetic fertilizers, particularly biostimulants and soil amendments. Biostimulants are natural products designed to enhance plant growth and productivity by improving nutrient use efficiency, increasing tolerance to abiotic stress, and strengthening plant immune systems [4,5,6]. Within this framework, the use of plant growth-promoting microbes (PGPM) has shown considerable potential to enhance nutrient uptake by stimulating root growth and architecture, as well as to protect crops from contaminants and pathogens [7,8]. Soil amendments are materials added to the soil to improve its physical properties, fertility, water and nutrient retention, and to support the growth of beneficial microbial communities [9]. Among them, biochar (Char), a carbon-rich material produced from agricultural or food waste via pyrolysis in a low-oxygen environment, has emerged as a sustainable and environmentally friendly option [10,11]. The combination of Char and PGPM has recently been investigated, observing a synergistic effect [4,12,13]. However, despite their increasing use, the effectiveness of these approaches in field conditions remains debated, and their impact on food quality and nutritional properties is not yet fully understood. This knowledge gap highlights the need for advanced scientific approaches capable of elucidating their effect at the molecular level.

In this context, metabolomics has emerged as a powerful tool for deciphering the biochemical effects of environmental conditions, specific treatments, and disease states. Metabolomics focuses on the comprehensive analysis of the metabolome, defined as the complete set of small molecules (typically <1500 Da) produced or modified through an organism’s biological processes [14]. As end products of the omics cascade, metabolites play a key role in cellular function and reflect changes occurring at the genomic, transcriptomic, and proteomic levels. Consequently, they are highly sensitive indicators of physiological alterations and serve as valuable biomarkers for assessing responses to treatments, environmental changes, and stress conditions. In addition, metabolomics has rapidly become a key methodology in nutrition and food research [4,14,15]. Advances in omics technologies have improved our understanding of nutritional complexity, particularly by enabling detailed characterization of the complex biochemical constituents of whole plant foods [16]. Furthermore, metabolomics allows the monitoring of metabolite changes during food processing, thereby supporting the production of higher-quality and more nutritionally consistent products [17]. Despite its potential, the comprehensive characterization of the metabolome remains challenging due to the diversity of metabolites in terms of molecular weight, polarity, volatility, and concentration levels. Consequently, no single analytical platform can achieve a complete metabolome coverage, requiring the integration of complementary analytical techniques [18].

The two main analytical platforms used in metabolomics are nuclear magnetic resonance spectroscopy (NMR) and high-resolution mass spectrometry (HRMS), each offering distinct advantages and limitations. NMR is characterized by high robustness, reproducibility, and quantification capabilities. It requires minimal sample preparation, is non-destructive, and provides valuable insights into metabolite structural elucidation, including the ability to distinguish compounds with identical molecular weights. However, NMR suffers from low sensitivity (in the micromolar-to-millimolar range) and signal overlap when complex matrices are analyzed [14,18]. Therefore, NMR is particularly well suited for the quantitative analysis of the most abundant metabolites present within the sample, including amino acids, carbohydrates and small carboxylic acids [18,19,20,21].

In contrast, HRMS offers very high sensitivity and selectivity, enabling the detection of thousands of metabolites at the nanomolar to picomolar level, and allowing tentative metabolite annotation through accurate mass measurements [14,18]. Beyond coupling of HRMS with chromatographic techniques as powerful instrumental platforms for untargeted metabolomics, the integration with ion mobility spectrometry (IMS) adds a quasi-orthogonal dimension of separation based on collisional cross-section (CCS). This additional separation enhances metabolite discrimination, thereby improving confidence in metabolite annotation [22,23].

Untargeted metabolomics generates large and complex datasets that require multivariate data analysis to extract meaningful biological information. In this context, chemometrics is required to harness the complexity of metabolomic datasets, enabling the identification of biologically relevant patterns. Furthermore, integrating the complementary capabilities of NMR and HRMS provides significant potential for the generation of more comprehensive chemical fingerprints [18]: this combined approach enhances metabolome coverage, improves metabolite identification and strengthens the robustness of biological interpretations [5,18].

In this context, data fusion (DF) strategies, aimed at integrating independently acquired metabolomics datasets, have emerged as powerful tools for investigating complex samples using complementary analytical techniques. Low- (LLDF), mid- (MLDF), and high-level (HLDF) data fusion strategies enable researchers to leverage the strengths of each analytical technique, improving model classification performance, and facilitating robust biomarker discovery [18,24]. In LLDF, datasets obtained from different analytical platforms are concatenated on a sample-wise basis to form a single data matrix [18,24,25]. This fused dataset is then subjected to supervised or unsupervised multivariate analysis. The major limitation of LLDF is the potential bias toward the data block with the highest covariance. To address this issue, MLDF involves the application of dimensionality reduction or feature extraction techniques to the original datasets prior to fusion. The resulting features are then concatenated into a new matrix for subsequent multivariate analysis, thereby reducing the imbalance caused by differences in dimensionality or variance between the original datasets [18,24,25]. Finally, HLDF entails building separate models for each data block and subsequently combining their outputs to generate a final prediction [24,25]. DF strategies have been applied to enhance classification performance in food authentication and quality control studies [18,25]. In particular, their application to merge datasets derived from complementary techniques such as HRMS and NMR holds great promise in the field of food metabolomics.

In a previous study, we demonstrated the capability of ultra-high performance liquid chromatography–ion mobility–high-resolution mass spectrometry (UHPLC-IMS-HRMS) to discriminate among different sustainable treatments applied to durum wheat samples (*Triticum durum*) [4]. In this study, proton nuclear magnetic resonance spectroscopy (^1^H-NMR) was applied to analyze the same set of samples, and the resulting dataset was integrated with HRMS data using both LLDF and MLDF approaches. In addition, due to their nutritional and technological relevance, gluten protein fractions, namely gliadins, high-molecular-weight glutenins (HMW-GS), and low-molecular-weight glutenins (LMW-GS), were integrated into the data fusion process [7]. Finally, the classification performance of the different data fusion strategies was compared in terms of classification accuracy, with particular emphasis on their impact on the reliability of biomarker discovery. This study highlights the growing importance of DF in metabolomics, demonstrating its ability to provide a more holistic view of biochemical processes in complex biological systems, including plant and food matrices.

## 2. Results and Discussion

### 2.1. UHPLC-IMS-HRMS Metabolomic Profile

**Figure 1:**
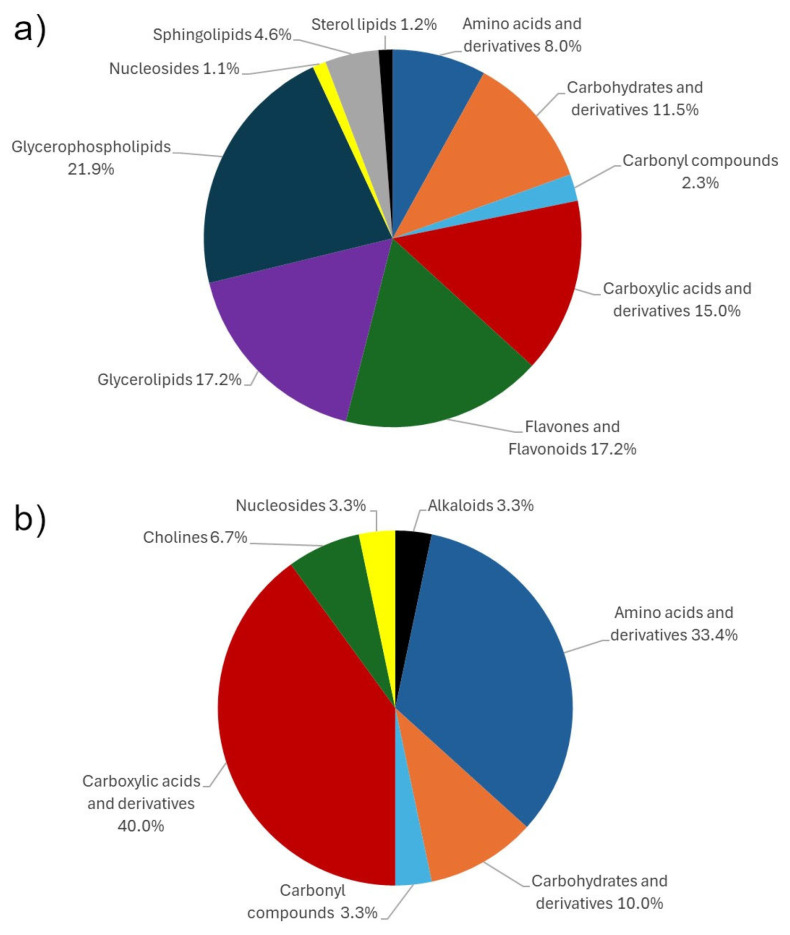
Pie charts summarizing the classes of annotated metabolites by (**a**) UHPLC-IMS-HRMS analysis, and (**b**) ^1^H-NMR analysis. The complete list of annotated metabolites by UHPLC-IMS-HRMS is reported in [4].

In our previous study, untargeted metabolomics based on UHPLC-IMS-HRMS was performed to investigate the metabolomic response of durum wheat to Char, PGPM and their combination (Char+PGPM) [4]. A total of 23,811 features were generated, subsequently filtered to 4686, considering intra-group variability (20%), minimum fold change compared to procedural blanks (≥3), and statistical power (≥0.8). The obtained dataset was investigated by multivariate data analysis by means of exploratory principal component analysis (PCA) and supervised partial least squares discriminant analysis (PLS-DA). Based on variable importance in progression (VIP) analysis, 285 features were extracted (VIP score ≥ 2), and submitted to the annotation process [4]. A total of 88 metabolites were annotated, mostly belonging to glycerophospholipids (21.8%), glycerolipids (17.2%), flavones and flavonoids (17.2%), and medium- to long-chain carboxylic acids and derivatives (14.9%), as reported in Figure 1a.

**Figure 2:**
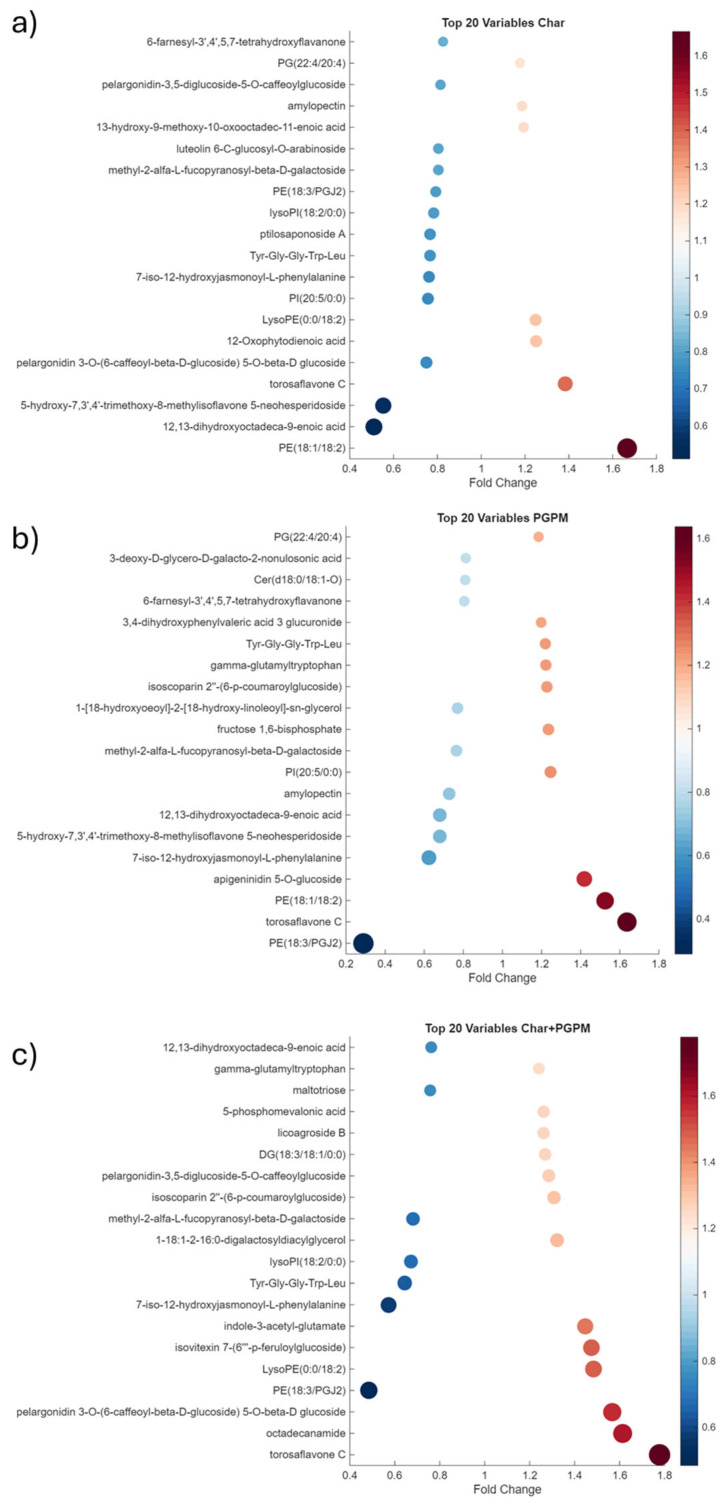
Representation of the 20 metabolites annotated by UHPLC-IMS-HRMS analysis that are most strongly up- or downregulated in response to each agro-sustainable treatment: (**a**) Char; (**b**) PGPM; (**c**) Char+PGPM. Both the color scale and dot size are proportional to the fold change in each treatment compared to CTRL samples. Fold change values > 1 indicate upregulation, while values < 1 indicate downregulation.

The effects of the applied treatments were assessed by calculating the fold changes for each treatment relative to the control (CTRL) samples. The 20 metabolites most affected by each treatment are shown in the scatterplot in Figure 2, highlighting the metabolic changes and their magnitude and direction of regulation. For each treatment, a specific metabolic signature was obtained.

The Char samples (Figure 2a) were characterized by a downregulation of metabolites belonging to the classes of lipids, phospholipids, glycerophospholipids, flavones and flavonoids and carboxylic acids and derivatives. The functions of some of these metabolites were associated with jasmonic acid (JA) metabolism. The recognized roles of JA are related to both growth regulation and gene expression regulation in response to biotic and abiotic stress [26]. The downregulation observed in the Char samples could be explained by considering that this amendment improves plant water status and reduces potential pathogens in the soil. In starch, lipids such as free fatty acids and lysophospholipids are integral components, while phospholipids, triglycerides, and glycolipids are present as surface components. The lipid content is positively associated with amylose concentration, and the resulting starch–lipid complexes can improve several technological properties of flour [27]. Other metabolites were also upregulated, including phosphatidylethanolamine PE (18:1/18:2) and phosphatidylglycerol PG (22:4/20:4), both involved in regulating membrane fluidity; 13-hydroxy-9-methoxy-10-oxooctadec-11-enoic acid, a complex fatty acid derivative with potential biological activities; and 12-Oxophytodienoic acid, a primary precursor of JA. A slight increase in amylopectin, which is a component of starch usually in a ratio 3:1 with respect to amylose, was also observed. The increase in the amylopectin content can impact both the technological properties of flours and the health of consumers [28].

The metabolic signature of the PGPM samples (Figure 2b) partially overlaps with that of Char samples and presents a set of downregulated metabolites, including PE (18:3/PGJ2), 12,13-dihydroxyoctadeca-9-enoic acid, the JA-derived metabolite 7-iso-12-hydroxyjasmonoyl-L-phenylalanine, multiple flavones, and the carbohydrate conjugate methyl-2-alfa-L-fucopyranosyl-beta-D-galactoside. The upregulated metabolites included different phospholipids, namely PG (22:4/20:4), PE (18:1/18:2), and phosphatidylinositol PI (20:5/0:0), as well as flavonoids, including torosaflavone C, isoscoparin 2″-(6-p-coumaroylglucoside), 3,4-dihydroxyphenylvaleric acid 3-glucuronide, and apigeninidin 5-O-glucoside. These compounds may contribute to flour color and bioactive properties [29]. The metabolite gamma-glutamyltryptophan, an amino acid derivative, was also upregulated in the PGPM samples. Although wheat gluten is characterized by a low level of tryptophan, gamma-glutamyltryptophan can be synthesized from glutamine and tryptophan by enzymes such as glutaminase, creating compounds with distinct flavor and health properties. Finally, fructose 1,6-bisphosphate upregulation may indicate enhanced carbohydrate metabolism.

Among the 20 most strongly influenced metabolites in the combined treatment (Figure 2c), only a few metabolites were downregulated, showing a partial overlap with those observed in Char samples. These included PE (18:3/PGJ2), 7-iso-12-hydroxyjasmonoyl-L-phenylalanine, methyl-2-alfa-L-fucopyranosyl-beta-D-galactoside, 12,13-dihydroxyoctadeca-9-enoic acid, Tyr-Gly-Gly-Trp-Leu, and lysoPI (18:2/0:0). Maltotriose levels were downregulated compared to CTRL samples, suggesting a reduced starch degradation. The Char+PGPM samples were characterized by a general upregulation of the metabolites compared to the other treatments [4]. In particular, these metabolites included 5-phosphomevalonic acid, an intermediate of the mevalonate pathway; licoagroside B, a saccharolipid signaling molecule that can be associated with abiotic stress response and in the regulation of plant growth, development, and defense against microbes; pelargonidin-3,5-diglucoside-5-O-caffeoylglucoside and pelargonidin 3-O-(6-caffeoyl-beta-D-glucoside) 5-O-beta-D-glucoside, which are involved in anthocyanin biosynthesis; 1-18:1-2-16:0-digalactosyldiacylglycerol, a galactolipid observed in plant membranes that can be associated with stress responses, like drought or high salinity; indole-3-acetyl-glutamate, a conjugated form of the hormone indole-3-acetic acid, that may serve as a temporary storage form [30]; isovitexin 7-(6‴-p-feruloylglucoside), which was primarily identified in barley leaves and other grains, and it is characterized by an antioxidant and anti-inflammatory activity [31]; and lysoPE (0:0/18:2), a glycolipid that can regulate plant growth. This set of upregulated metabolites suggests a beneficial effect of the combined treatment in terms of (i) ability to face abiotic stress through improved membrane integrity; (ii) improved seed quality via increased anthocyanin biosynthesis; (iii) increased antioxidant properties of flour; and (iv) improved technological quality due to upregulation of several lipids and glycolipids, which are key determinants of bread quality [27]. In addition, the anthocyanin biosynthesis may influence seed color, together with elevated levels of 5-phosphomevalonic acid, indicative of enhanced activity of the mevalonate pathway [32]. This pathway is involved in the biosynthesis of secondary metabolites, including volatile compounds that are also associated with flour quality.

### 2.2. ^1^H-NMR Metabolomic Profile

A total of 279 features were obtained when ^1^H-NMR analysis was performed. The removal of noise and solvent signal, along with signal integration, resulted in 116 features, leading to the annotation of 30 compounds. It has to be considered that, unlike HRMS, where a single feature is related to a single compound, multiple peaks and signals in ^1^H-NMR can be associated with a metabolite, thus strongly limiting the number of annotated compounds [33]. This highlights the challenges inherent to NMR-based metabolite annotation, particularly in complex biological matrices: extensive peak overlap, signal suppression, and intrinsically lower sensitivity collectively reduce spectral resolution and complicate the annotation of minor components. As depicted in Figure 1b, the annotated metabolites are classified mainly as short-chain carboxylic acids (40%) or amino acids and derivatives (33.3%). It is worth noting that the annotated compounds could not be detected by UHPLC-IMS-HRMS, since small polar compounds eluted early under the applied conditions, resulting in insufficient retention and poor detectability. In addition, co-elution with highly concentrated matrix components may occur, resulting in pronounced matrix effects. By contrast, short-chain fatty acids and amino acids are particularly amenable to NMR analysis. These findings highlight the substantial synergy between the two platforms and support the use of data fusion strategies to improve the metabolome profiling and investigate the impact of the investigated treatments.

**Figure 3:**
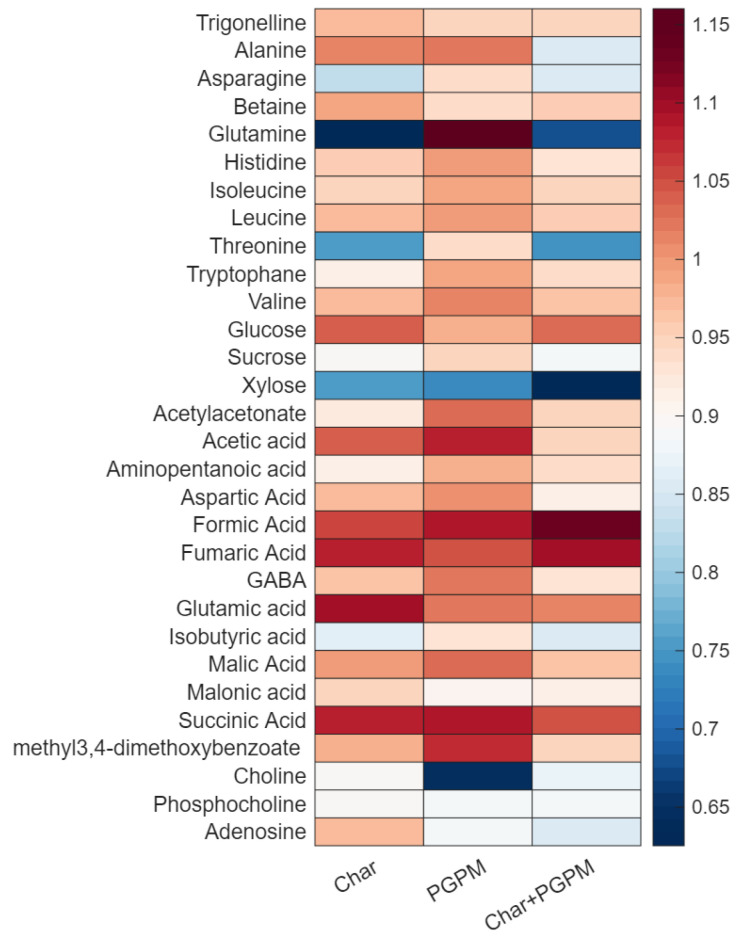
Heatmap of ^1^H-NMR-annotated metabolites across agro-sustainable treatments. Rows represent annotated metabolites, and columns represent treatments (Char, PGPM, and Char+PGPM). The color intensity reflects fold change values relative to CTRL samples, with red indicating upregulation and blue indicating downregulation.

Figure 3 provides a comprehensive overview of the metabolite modulation annotated by ^1^H-NMR analysis. As reported in the figure, a distinct pattern of relative abundance was obtained for each treatment considering the fold change ratio of the median response related to each treatment compared to the CTRL conditions.

Regarding amino acids, the application of either Char or PGPM resulted in alanine levels comparable to those of the CTRL group, while a slight downregulation was observed for the Char+PGPM treatment. Consequently, the alanine degradation process to lactate may be reduced in wheat treated with both Char and PGPM. The level of asparagine was decreased in Char and Char+PGPM samples, potentially reducing the formation of acrylamide in processed foods [34]. Glutamine was downregulated in Char and Char+PGPM samples and slightly upregulated in PGPM, whereas the level of glutamic acid was increased in Char samples. These amino acids are strictly related to the process of N assimilation in wheat grains through the glutamine synthetase and glutamate synthase cycle [35]. The observed variations suggest that the applied agro-sustainable treatments could affect ammonium assimilation into seed proteins. The level of threonine in wheat extracts from Char or Char+PGPM showed a significant decrease. The pool of free amino acids plays a key role in the synthesis of secondary metabolites, which are involved in defense responses during seed germination [36].

As for sugars, glucose, sucrose, and xylose were characterized by very different profiles: glucose presented similar levels for all treatments, whereas sucrose levels were reduced in Char and Char+PGPM samples; xylose was downregulated in all treatments, particularly in Char+PGPM samples. These carbohydrates have different roles in the seeds: wheat flour naturally contains small amounts of sucrose, glucose is mainly stored in starch, and xylose is a component of arabinoxylan fibers, which can become available upon microbial fermentation. Furthermore, the pathways of cytosolic glycolysis and the TCA cycle were affected by the applied treatments considering the general upregulation of succinic, fumaric, and malic acids compared to CTRL samples. 3,4-Dihydroxybenzoate is naturally present in whole wheat, acting as an antioxidant, and it was upregulated in PGPM samples.

It is noteworthy that the levels of choline exhibit a significant decrease in wheat extracts that have been treated with PGPM, suggesting a growth deficiency. Phosphocholine showed slight downregulation compared to CTRL samples. This phospholipid is concentrated in the germ and aleurone layers in wheat seeds. It can affect technological properties since it improves the ability of the dough to hold gas (foam stability) through interactions with glycolipids [37].

### 2.3. Data Fusion Analysis

Considering the different chemical classes annotated using HRMS and ^1^H-NMR, DF was performed to jointly exploit the complementary information provided by the two platforms. This strategy allows for a more integrated interpretation of metabolite profiles, capturing a broader range of compounds involved in biological pathways, and improving the performance and robustness of the resulting models. In addition to the metabolomic profiles obtained by ^1^H-NMR and HRMS, gluten protein fractions were incorporated into the data fusion framework due to their nutritional and technological relevance. Specifically, gliadins, HMW-GS, and LMW-GS were included, allowing the fused dataset to capture not only the metabolite composition, but also protein features that play a key role in the quality and functional properties of wheat [38].

Both LLDF and MLDF data fusion modeling were applied to the datasets. As described in materials and methods, LLDF was carried out by merging the considered data sources and performing a PLS-DA analysis, while MLDF was performed by concatenating the first 5 PC scores calculated separately for each data block. The obtained model performance metrics evaluated using the external test set are reported in Table 1.

The analysis of each analytical block revealed that the UHPLC-IMS-HRMS and protein datasets exhibited good discrimination capability, with NER values of 0.98 and 0.90, respectively. In contrast, the single ^1^H-NMR block exhibited poor classification performance (NER: 0.65). This behavior can be attributed to the lower sensitivity of NMR in detecting minor variations in metabolites and the more complex, overlapping signals typical of NMR spectra, which reduce its ability to discriminate among sample classes [18]. When all the blocks were considered, LLDF and MLDF yielded similar classification performance metrics. However, MLDF was preferred because it provided a better balance of information contribution from each data block, particularly considering the large differences in the number of original features (88, 30, and 3 for HRMS, ^1^H-NMR, and protein blocks, respectively). In fact, one major limitation of LLDF is its potential bias toward the data block with the largest covariance.

### 2.4. Pattern Analysis of Fused Metabolomic Data

**Figure 4:**
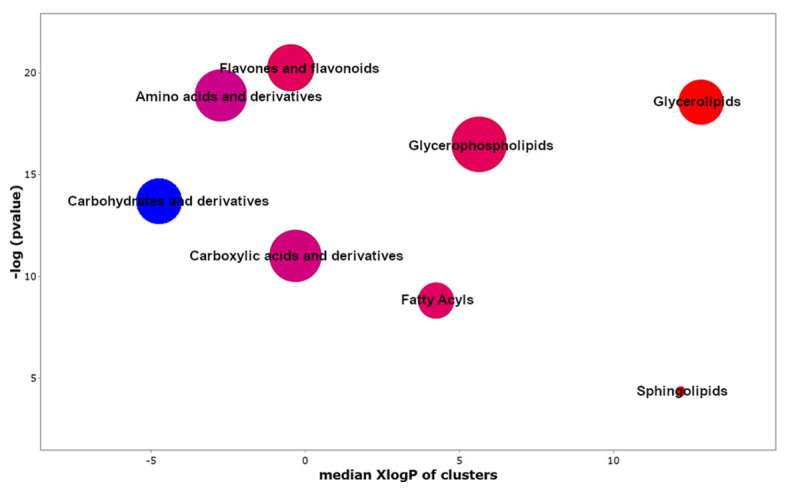
Bubble plot summarizing metabolite class enrichment considering Char+PGPM treatment. Each bubble corresponds to a metabolite class, with bubble size indicating the number of metabolites within each class. The color intensity reflects fold change values relative to CTRL samples, with red indicating upregulation and blue indicating downregulation.

By applying the Chemical Similarity Enrichment Analysis (ChemRICH) approach [39], the annotated compounds were merged into structurally related clusters, allowing the identification of enrichment patterns even for metabolites with limited or missing pathway annotations. Several clusters significantly enriched in the samples treated with both Char and PGPM were observed, indicating coordinated changes within specific chemical classes (Figure 4). Clusters positioned higher on the *y*-axis, namely flavones and flavonoids, amino acids and derivatives, and glycerolipids, showed the strongest statistical significance, indicating coordinated alterations within these groups. The distribution along the *x*-axis (median XlogP) suggested that both hydrophilic clusters (e.g., carbohydrates and carboxylic acids and derivatives) and more hydrophobic clusters (e.g., glycerolipids and glycerophospholipids) were affected, suggesting that the combined treatment impacted metabolites across a broad polarity range. Flavones and flavonoids emerged as one of the most significantly enriched classes, supporting an increased contribution of secondary metabolism, potentially linked to grain quality traits or stress-related responses [40]. Notably, lipid-related clusters including glycerolipids, glycerophospholipids, and, to a lesser extent, sphingolipids also showed distinct enrichment, consistent with alterations in membrane-associated or energy-related lipid metabolism.

**Figure 5:**
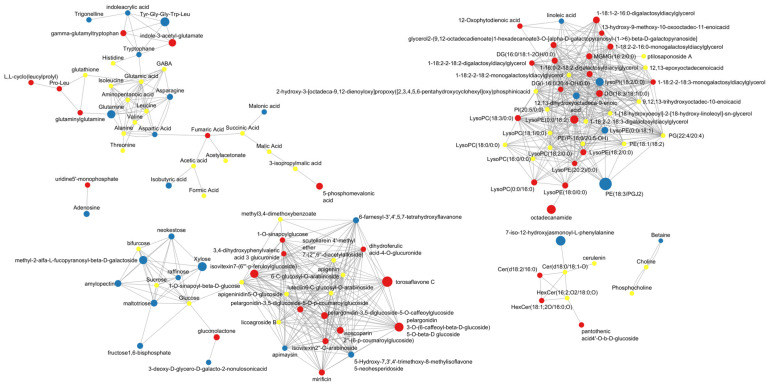
MetaMapp network of annotated metabolites: node colors indicate regulation patterns across the identified metabolites (red: upregulated, blue: downregulated, yellow: marginal or no change; dot size is proportional to the fold change) considering the combined treatment Char+PGPM compared to CTRL samples.

Finally, coordinated metabolic changes were investigated in terms of up- and downregulation considering the combined Char+PGPM treatment compared to CTRL (Figure 5). The connectivity within this cluster indicates that these changes involve structurally related compounds sharing common biosynthetic origins. Upregulation was observed within the polyphenol cluster, particularly among flavonoid glycosides and related phenolic compounds, suggesting enhanced accumulation of secondary metabolites, potentially associated with antioxidant capacity and stress response in the wheat grains [41]. Various flavones and flavonoid compounds were upregulated after the use of combined treatment, including 1-sinapoylglucose, scutellarein 4′-methyl ether, dihydroferulic acid 4-O-gluconoride, torosaflavone C, and isovitexin derivatives. Flavones and flavonoids play multifunctional roles in plant physiology, acting as antioxidants, signaling molecules, and defense compounds [42]. The observed metabolic changes are consistent with increasing evidence that PGPM can stimulate secondary metabolite production in plants by activating systemic signaling and defense pathways [43]. Glycosylation generally enhances the stability and solubility of flavonoids, as well as their transport and storage within plant tissues. Representatives of pelargonidin glycosides related to anthocyanin biosynthesis, including pelargonidin-3,5-diglucoside derivatives with coumaroyl and caffeoyl moieties, were also upregulated [44,45]. The upregulation of compounds belonging to this cluster is consistent with previous findings regarding the inoculation of PGPM as a sustainable treatment [43,46].

The northeast cluster is characterized by a general upregulation of carboxylic acid derivatives, glycolipids, and phospholipids in durum wheat grains treated with both PGPM and Char. This pattern suggests a treatment-induced modulation of carbon–lipid metabolism, likely reflecting enhanced metabolic activity and membrane remodeling in response to improved nutrient availability and plant–microbe interactions. This behavior has been previously reported in the literature, as PGPM are known to influence central carbon metabolism through improved nutrient uptake [47], while Char can alter soil physicochemical properties and microbial activity [48,49]. Within this cluster, the upregulation of glycolipids—particularly galactolipids—could be associated with starch biosynthesis [50]. In particular, the accumulation of LysoPEs is often associated with enhanced membrane remodeling and signaling activity [51] and may reflect an increase in phospholipase activity induced by PGPM. The observed upregulation of diacylglycerols (DGs) can be related to lipid degradation and biosynthetic pathways: DG accumulation has been associated with signaling cascades activated by environmental and biological stimuli [52]. The upregulation of glycolipids and phospholipids could also be associated with the increased abundance of LMW-GS present in the samples subjected to the combined PGPM and Char treatment, as these proteins are mostly associated with glutenins in wheat grains via hydrogen bonding and hydrophobic interactions [53].

In contrast, several carbohydrate-related metabolites, including mono- and oligosaccharides, are predominantly downregulated, suggesting a possible redistribution of carbon resources during grain development or maturation. This trend may reflect altered starch biosynthesis or carbohydrate turnover, consistent with the central role of carbon allocation in determining grain composition and quality [54]. Organic acids involved in central carbon metabolism show more moderate regulation, with a mixture of up- and downregulated nodes. Lipid metabolism also shows distinct regulation patterns, with specific lipid subclasses, such as lysophospholipids and glycerolipids, exhibiting upregulated nodes, suggesting membrane remodeling or changes in lipid storage [55].

Amino acids and related metabolites exhibit heterogeneous behavior: most of the detected compounds remained unchanged, whereas Pro-Leu, glutaminylglutamine, gamma-glutamyltryptophan and L,L-cyclo(leucylprolyl) are upregulated in the combined treated samples, and tryptophan, asparagine, glutamine, and betaine are underexpressed.

The network also shows downregulation of carbohydrate metabolites, including neokestose, xylose, raffinose, methyl-2-α-fucopyranosyl-β-D-galactoside, amylopectin, maltotriose, and fructose-1,6-bisphosphate in samples treated with the combined approach. This reflects alterations in carbon partitioning and energy metabolism within the developing grain. Oligoccharides and reserve polysaccharides, such as raffinose, neokestose, maltotriose, and amylopectin, are major components of carbohydrate storage pools in cereal grains, playing a key role in carbon storage, osmotic regulation, and stress responses [56].

## 3. Materials and Methods

### 3.1. Chemicals and Materials

The HPLC-grade methanol used for extraction was purchased from VWR Chemicals International (Radnor, PA, USA). The sodium phosphate for the NMR buffer was obtained from Roth (Karlsruhe, Germany); the D_2_O from Deutero (Kastellaun, Germany); and the TMSP-d_4_ standard was from Merck (Darmstadt, Germany). Double-distilled water was used for all steps.

### 3.2. Sample Preparation for ^1^H-NMR Profiling

Durum wheat (*Triticum durum Desf*., cultivar Svevo) was cultivated during in field trials conducted in 2021 as previously reported [4]. Treatments included the application of Char, PGPM, and Char+PGPM, alongside the CTRL condition, consisting of a sample without organic fertilization. For each treatment group, 9 independent samples were collected, for a total of 36 samples. Wheat flour extraction was performed according to the protocol reported by Riboni et al. (2023) [4], with minor modifications. Briefly, 50 mg of sample was extracted with 1 mL of a methanol/water solution (70/30 *v*/*v*) by sonicating the sample at 15 °C for 30 min. Then the sample was centrifuged at 14.000× *g* for 10 min at 4 °C and 800 µL of the supernatant was collected. The extract was dried under vacuum for 5 h using the Concentrator plus system (Eppendorf, Hamburg, Germany). The dried sample was reconstituted in 40 µL of 100% D_2_O phosphate buffer and 0.5 mM of the internal TMSP-d_4_-standard (3 (trimethylsilyl)propionic-2,2,3,3-d_4_). A volume of 40 µL of each sample was transferred to 1.7 mm NMR tubes (Bruker, Billerica, MA, USA) for ^1^H-NMR analysis.

### 3.3. ^1^H-NMR Profiling

The ^1^H-1D spectra were acquired using TopSpin version 4.5.0 on a Bruker AV NEO 600 MHz spectrometer (Bruker, Billerica, MA, USA) equipped with a 1.7 mm QCI cryoprobe (^1^H [^13^C, ^15^N]) at 298 K. The NMR data were collected using a SampleJet automated sample changer with an automatic tune and match system and using ICON NMR 6.2.2 software (Bruker, Billerica, MA, USA). The ^1^H-1D spectra were acquired with a spectral width of 19.8 ppm, 95 K data points, 512 scans, 16 dummy scans and a relaxation time of 1 s using the noesygppr1d pulse program with water presaturation. The processing of the NMR spectra (Fourier transformation and phase correction) was conducted using TopSpin version 4.5. The subsequent analysis was performed using MNova version 15.01.35756 (Mestrelab Research, Santiago, Spain), with baseline correction and reference to the TMSP-d_4_ peak at δ = 0.00 ppm. The Chenomx Profiler (NMR suite software version 9.0, Edmonton, AB, Canada) and the human metabolome database (HMDB) were used for peak identification. The spectra were analyzed with a 0.04 ppm binning across a chemical-shift window of –0.36 to 10.81 ppm.

### 3.4. UHPLC-IMS-HRMS and Gluten Protein Data

The dataset obtained from untargeted UHPLC-IMS-HRMS metabolomics was reported in Riboni et al. (2023) [4]. Briefly, data-independent acquisition was performed in both positive and negative ion mode using electrospray ionization. The resulting features were processed using PLS-DA, and data reduction was performed considering only those features having a variable importance in projection scores ≥ 2. Compound annotation was performed by comparing the obtained spectra with those stored in online libraries, using a mass error tolerance of 5 ppm for precursor ions and 10 ppm for fragment ions. An isotope similarity threshold of 80% was set. Experimental CCS values were compared with those predicted using the ALLCCS2 algorithm [57] to confirm the previous annotation. A total of 177 features were obtained, of which 88 were annotated at levels 1–3 [58].

The protein content in terms of gliadins, HMW-GS, and LMW-GS was obtained as reported in Caldara et al. 2024 [7].

### 3.5. Data Modeling and Fusion Strategies

The samples were distributed in four equally represented classes: the datasets consisted of 36 samples with 30, 88 and 3 variables for the NMR, HRMS and protein datasets, respectively. The data structure was initially analyzed using PCA, a widely recognized multivariate technique for exploratory data analysis [59]. PLS-DA was used as a multivariate classification method to discriminate the four classes. PLS-DA is based on the PLS2 algorithm [59]. In LLDF, datasets obtained from ^1^H-NMR, HRMS, and protein datasets were concatenated sample-wise to form a single matrix, with rows representing samples and columns representing features obtained from each technique [18,24,25]. The fused training data were then used to build the PLS-DA classification model. The importance of each variable was assessed by Wilks’ lambda (Table S1) [60], which is a statistic that estimates the discriminatory power of variables based on the ratio of within-group to between-group sums of squares, representing the within-class and between-class variability, respectively. Wilks’ lambda ranges from 0 to 1, with values close to 0 indicating that class means differ substantially and, therefore, that the variables have strong discriminatory capability between classes. MLDF was applied, performing separately PCA on the ^1^H-NMR and HRMS blocks, selecting 5 PCs. PCA scores for the test samples were obtained by projecting them onto the PCA models derived from the training set. Then the blocks of the score matrices from both ^1^H-NMR and HRMS were combined with the gluten protein dataset, resulting in a combined score matrix constituted by 13 columns, which was subsequently used to train the PLS-DA classification model.

### 3.6. Model Validation

Due to the relatively small size of the datasets, models were validated through a double validation procedure [61] based on 1000 iterations. In each iteration, samples were randomly split into training (80% of the samples) and test (20%) sets, while maintaining class proportion. Each individual data block (HRMS, ^1^H-NMR, and protein) was independently analyzed using PLS-DA to evaluate classification performance and used to carry out LLDF and MLDF. The optimal number of latent variables for each model was determined through Venetian-blind cross-validation (4 groups) performed on the training set, using the NER (also known as balanced accuracy, which is the average of class sensitivities) as the optimization criterion. The resulting PLS-DA models were then used to predict class membership of the test samples. The classification performance of individual data blocks, as well as of the LLDF and MLDF models, was estimated considering NER, class precision (the ratio between the number of samples belonging to a class correctly predicted and the total number of samples predicted in that class), sensitivity (ratio of samples of a given class that are correctly predicted to all true samples of that class) and specificity (ratio of samples belonging to other classes that are correctly predicted as not belonging to the given class) as figures of merit [62].

### 3.7. Software

Data analysis was performed using Matlab ver. R2025b, using a homemade algorithm based on the PCA toolbox [63] and the classification toolbox [64]. The ChemRICH analysis was applied to characterize the effect of the investigated agro-sustainable treatments on the metabolomic profile [38] and the MetaMapp [65] online tool was applied to highlight coordinated metabolic changes.

## 4. Conclusions

In this study, untargeted metabolomics based on UHPLC-IMS-HRMS and ^1^H-NMR proved effective in investigating the effects of sustainable agrifood treatments on durum wheat. The complementarity of the two techniques was demonstrated, enabling the detection of 88 metabolites—primarily glycerophospholipids, glycerolipids, flavones and flavonoids, and medium- to long-chain carboxylic acids and their derivatives by UHPLC-IMS-HRMS—and 33 compounds, mainly short-chain carboxylic acids and amino acids and their derivatives, by ^1^H-NMR. The strong complementarity between the two analytical platforms underscores the value of the combined approach for achieving comprehensive metabolome profiling. Both LLDF and MLDF strategies were successfully applied to the combined datasets: MLDF achieved superior classification performance and a more balanced contribution of information from each data block, particularly considering the substantial differences in the number of original features.

The analysis of the fused dataset improved the biological interpretation of the effects of sustainable treatments on wheat, highlighting changes in bioactive compounds that may contribute to enhanced plant performance, with potential benefits for consumer health and technological applications. The integrated Char+PGPM treatment induced upregulation of flavones and flavonoids, and lipid-related classes, indicating a broad metabolic reprogramming affecting both hydrophilic and hydrophobic metabolites. In particular, the strong enrichment of flavonoids and anthocyanin-related compounds suggested enhanced secondary metabolism, potentially linked to improved stress responsiveness, antioxidant capacity, and grain quality. Lipid-related clusters, including glycerolipids and glycerophospholipids, were also markedly affected, consistent with membrane remodeling, altered carbon–lipid metabolism, and signaling processes. Conversely, the downregulation of several carbohydrate metabolites and storage polysaccharides suggested a redistribution of carbon resources during grain growth.

Finally, the results achieved in this study strengthen the potential of Char+PGPM sustainable treatment not only to improve plant growth and stress resilience but also to promote the biosynthesis of secondary metabolites having noticeable antioxidant activity and nutritional properties, in accordance with the goals of sustainable agriculture and crop biofortification strategies.

## Figures and Tables

**Table 1:** Model performance metrics: non-error rate (NER), precision (pre), sensitivity (sn), and specificity (sp) calculated on the external test set.

			CTRL			Char			PGPM			Char+PGPM		
Source	Fusion	NER	pre	sn	sp	pre	sn	sp	pre	sn	sp	pre	sn	sp
HRMS	single block	0.98	0.99	0.95	1.00	0.96	0.99	0.98	1.00	0.98	1.00	0.98	1.00	0.99
^1^H-NMR	single block	0.65	0.73	0.61	0.92	0.63	0.50	0.90	0.64	0.66	0.88	0.63	0.84	0.83
proteins	single block	0.90	0.99	0.74	1.00	1.00	0.99	1.00	0.76	1.00	0.90	0.94	0.89	0.98
HRMS + ^1^H-NMR + protein	LLDF	0.99	1.00	0.99	1.00	0.99	0.98	1.00	0.97	0.99	0.99	0.99	1.00	1.00
HRMS + ^1^H-NMR + protein	MLDF	0.99	1.00	1.00	1.00	1.00	1.00	1.00	1.00	0.98	1.00	0.98	1.00	0.99

## Data Availability

Data will be made available on request.

## Supplementary Materials

The following supporting information can be downloaded at https://www.mdpi.com/article/10.3390/molecules31060922/s1, Table S1: Wilk’s lambda of the annotated features.

## Abbreviations

The following abbreviations are used in this manuscript:

CCS	collisional cross-section
Char	biochar
Char+PGPM	biochar and plant growth-promoting microbes combined treatment
CTRL	control treatment
DF	data fusion
DH	diacylglycerol
^1^H-NMR	proton nuclear magnetic resonance spectroscopy
HLDF	high-level data fusion
HMW-GS	high-molecular-weight glutenins
HRMS	high-resolution mass spectrometry
IMS	ion mobility spectrometry
JA	jasmonic acid
LLDF	low-level data fusion
LMW-GS	low-molecular-weight glutenins
MLDF	mid-level data fusion
NER	non-error rate
NMR	nuclear magnetic resonance spectroscopy
PCA	principal component analysis
PE	phosphatidylethanolamine
PG	phosphatidylglycerol
PGPM	plant growth-promoting microbes
PI	phosphatidylinositol
PLS-DA	partial least squares discriminant analysis
pre	precision
sn	sensitivity
sp	specificity
UHPLC-IMS-HRMS	ultra-high performance liquid chromatography–ion mobility–high-resolution mass spectrometry
VIP	variable importance in progression
