# Supplementary material for: Data Fusion Combining High-Resolution Mass Spectrometry and 1H-NMR Metabolomic Data with Gluten Protein Content to Assess the Impact of Agro-Sustainable Treatments on Durum Wheat
Source: Molecules. 2026 Mar 10;31(6):922. doi: 10.3390/molecules31060922 (PMC13029761; doi:10.3390/molecules31060922)
Supplement: Supplementary file 1 [file molecules-31-00922-s001.zip › molecules-4151545-supplementary.pdf]

Supplementary Material

Data Fusion Combining High-Resolution Mass Spectrometry and ^{1}H-NMR Metabolomic Data with Gluten Protein Content to Assess the Impact of Agro-Sustainable Treatments on Durum Wheat

Nicolò Riboni [1,*], Enmanuel Cruz Muñoz [2], Christina Muhs [3], Monica Mattarozzi [1], Marina Caldara [1], Sara Graziano [1], Christian Richter [3], Harald Schwalbe [3], Nelson Marmiroli [1], Davide Ballabio [2], Mariolina Gullì [1,*], Maria Careri [1] and Federica Bianchi [1,*]

Corresponding authors:
N.R.: nicolo.riboni@unipr.it; +39-0521-902174
M.G: mariolina.gulli@unipr.it; +39-0521-905486
F.B.: federica.bianchi@unipr.it, +39-0521-905446

Table S1. Wilk's lambda of the annotated features

Feature	Wilk's lambda
gliadins	0.05
12,13-dihydroxyoctadeca-9-enoic acid	0.18
7-iso-12-hydroxyjasmonoyl-L-phenylalanine	0.20
5-hydroxy-7,3',4'-trimethoxy-8-methylisoflavone 5-neohesperidoside	0.21
maltotriose	0.21
amylopectin	0.25
lysoPE(0:0/18:2)	0.26
raffinose	0.27
neokestose	0.32
bifurcose	0.38
torosaflavone C	0.38
13-hydroxy-9-methoxy-10-oxooctadec-11-enoic acid	0.40
LMW-GS	0.40
HMW-GS	0.41
xylose	0.45
methyl-2-alfa-L-fucopyranosyl-beta-D-galactoside	0.46
Cer(d18:0/18:1-O)	0.46
DG(18:3/18:1/0:0)	0.46
lysoPE(18:2/0:0)	0.46
lysoPE(20:2)/0:0)	0.46
choline	0.47
octadecanamide	0.48
luteolin 6-C-glucosyl-O-arabinoside	0.48
isovitexin 2''-O-arabinoside	0.48
lysoPE(18:0/0:0)	0.49
apimaysin	0.50
lysoPC(0:0/16:0)	0.51
dihydroferulic acid 4-O-glucuronide	0.53
mirificin	0.53
1-18:2-2-18:3-monogalactosyldiacylglycerol	0.53
12-oxophytodienoic acid	0.54
uridine 5'-monophosphate	0.54
ptilosaponoside A	0.55
Tyr-Gly-Gly-Trp-Leu	0.56
lysoPC(18:3/0:0)	0.56
gluconolactone	0.56
succinic acid	0.57
pelargonidin 3-O-(6-caffeoyl-beta-D-glucoside) 5-O-beta-D glucoside	0.58
fructose 1,6-bisphosphate	0.58
HexCer(18:1;2O/16:0;O)	0.59
PE(18:3/PGJ2)	0.59
3-isopropylmalic acid	0.59
3-deoxy-D-glycero-D-galacto-2-nonulosonic acid	0.59
6-farnesyl-3',4',5,7-tetrahydroxyflavanone	0.59
indole-3-acetyl-glutamate	0.59

gamma-glutamyltryptophan	0.59
DG(16:0/18:1-2OH/0:0)	0.60
tryptophane	0.60
12,13-epoxyoctadecenoic acid	0.61
Cer(d18:2/16:0)	0.61
adenosine	0.61
formic acid	0.61
Pro-Leu	0.61
lysoPI(18:2/0:0)	0.61
PG(22:4/20:4)	0.61
1-18:1-2-16:0-digalactosyldiacylglycerol	0.62
1-O-sinapoylglucose	0.62
pelargonidin-3,5-diglucoside-5-O-p-coumaroylglucoside	0.62
cerulenin	0.62
indoleacrylic acid	0.63
9,12,13-trihydroxyoctadec-10-enoic acid	0.63
lysoPC(18:0/0:0)	0.63
5-phosphomevalonic acid	0.63
1-18:2-2-18:3-digalactosyldiacylglycerol	0.63
lysoPC(16:0/0:0)	0.64
1-18:2-2-16:0-monogalactosyldiacylglycerol	0.64
isoscoparin 2''-(6-p-coumaroylglucoside)	0.64
apigenin 6-C-glucosyl-O-arabinoside	0.64
acetic acid	0.65
isovitexin 7-(6'''-p-feruloylglucoside)	0.65
DG(i-16:0/20:4-OH/0:0)	0.65
3,4-dihydroxyphenylvaleric acid 3 glucuronide	0.65
linoleic acid	0.65
PI(20:5/0:0)	0.65
MGMG(18:2/0:0)	0.66
apigeninidin 5-O-glucoside	0.66
licoagroside B	0.66
glucose	0.66
1-18:2-2-18:2-digalactosyldiacylglycerol	0.66
glutathione	0.66
pelargonidin-3,5-diglucoside-5-O-caffeoylglucoside	0.67
lysoPC(18:2/0:0)	0.67
1-O-sinapoyl-beta-D-glucose	0.68
glycerol 2-(9,12-octadecadienoate) 1-hexadecanoate 3-O-[alpha-D-galactopyranosyl-(1->6)-beta-D-galactopyranoside]	0.68
HexCer(16:2;O2/18:0;O)	0.68
L,L-cyclo(leucylprolyl)	0.69
1-18:2-2-18:2-monogalactosyldiacylglycerol	0.69
scutellarein 4'-methyl ether 7-(2'',6''-diacetylalloside)	0.70
pantothenic acid 4'-O-b-D-glucoside	0.70
glutaminylglutamine	0.70
1-16:0-2-18:2-digalactosyldiacylglycerol	0.70

glutamine	0.71
threonine	0.72
glutamic acid	0.74
lysoPC(18:1/0:0)	0.74
fumaric acid	0.76
lysoPE(0:0/18:1)	0.77
aspartatic acid	0.79
2-hydroxy-3-[octadeca-9,12-dienoyloxy]propoxy([2,3,4,5,6-pentahydroxycyclohexyl]oxy)phosphinic acid	0.79
PE(18:1/18:2)	0.80
1-[18-hydroxyoeoyl]-2-[18-hydroxy-linoleoyl]-sn-glycerol	0.80
GABA	0.81
aminopentanoic acid	0.81
PE(P-16:0/20:5-OH)	0.81
malonic acid	0.82
isobutyric acid	0.82
sucrose	0.85
betaine	0.87
malic acid	0.89
trigonelline	0.90
asparagine	0.91
alanine	0.92
leucine	0.92
acetylacetonate	0.92
histidine	0.94
methyl-3,4-dihydroxybenzoate	0.94
phosphocholine	0.96
valine	0.96
isoleucine	0.97
